# Experience in the Application of Augmented Reality Technology in the Surgical Treatment of Patients Suffering Primary and Recurrent Pelvic Tumors

**DOI:** 10.3390/jpm14010019

**Published:** 2023-12-22

**Authors:** Vladimir M. Ivanov, Anton M. Krivtsov, Anton Yu. Smirnov, Vladimir G. Grebenkov, Dmitry A. Surov, Michail S. Korzhuk, Sergey V. Strelkov, Elena G. Ivanova

**Affiliations:** 1 Higher School of Theoretical Mechanics, Peter the Great St. Petersburg Polytechnic University, 195251 Saint Petersburg, Russia; anton.krivtsov@spbstu.ru (A.M.K.); ishunpo0@gmail.com (A.Y.S.); egi1955@yandex.ru (E.G.I.); 2 Naval Surgery Chair, S. M. Kirov Military Medical Academy, 194044 Saint Petersburg, Russia; grebenkov_89@mail.ru (V.G.G.); sda120675@mail.ru (D.A.S.); gensurg@mail.ru (M.S.K.); 3 Coloproctology Department, Saint-Petersburg I. I. Dzhanelidze Research Institute of Emergency Medicine, 192242 Saint Petersburg, Russia; 4 N.N. Petrov National Medical Research Center of Oncology, 197758 Saint Petersburg, Russia; 5 Flinders Street Campus, Torrens University, Melbourne, VIC 3000, Australia; sergin3d2d@gmail.com

**Keywords:** augmented reality, combined surgeries, locally spread tumors, preoperative planning, recurrent tumors, pelvic evisceration

## Abstract

Surgical treatment of locally spread tumors in pelvic organs remains an urgent and complicated oncological problem. The recurrence rate after radical treatment ranges from 15.1% to 45.2%. The key to successful and safe surgical intervention lies in meticulous planning and intraoperative navigation, including the utilization of augmented reality (AR) technology. This paper presents the experience of clinically testing an AR technology application algorithm in the surgical treatment of 11 patients. The main stages of the algorithm are described. Radical operations incorporating intraoperative AR technology with favorable outcomes were performed on eight patients. One patient underwent a palliative intervention, while two patients did not undergo surgery. The testing of the algorithm for the application of AR technology in the surgical treatment of primary and recurrent pelvic tumors demonstrated both a technical possibility and reproducibility of this algorithm and the AR technology itself in clinical practice.

## 1. Introduction

The morbidity of pelvic organ malignancies in Russia has shown a steadily increasing trend over the past decade. Neoplasms of the rectum, bladder, ovaries, vagina, uterine body, and cervix account for up to 26% of the total structure of malignancies [1]. According to various authors, about 18–32% of newly identified malignant neoplasms of the pelvic organs are locally widespread. This extent of spread can lead to the refusal of radical surgery and the prescription of palliative therapy, resulting in a total life expectancy of 1 to 6 months [2,3,4].

Modern advancements in diagnostics, surgical techniques, and complex chemoradiotherapy now permit the performance of R0-resections for tumors with massive invasion into the organs and anatomical formations of the pelvis. Despite these advancements, the frequency of local recurrences after such operations is still 15.1–45.2%, and the overall five-year survival rate varies from 23.0 to 75.0%. This rate largely depends on the morphological structure of the tumor [5,6]. For patients with recurrent tumors, a full range of treatment options is considered. The frequency of performing radical resections for recurrent tumors of the pelvic organs (RTPO) is 54.0–66.0% [5,7,8,9].

The necessity to radically remove a malignant neoplasm, including the excision of neighboring organs and anatomical structures involved in the malignant process, underscores the importance of comprehensive patient examination. One of the most crucial cornerstones of successful and safe surgical intervention is its careful planning, which is based on the analysis of data from the patient’s instrumental examination. Planning implies a detailed preoperative discussion of the steps of the intervention and, if possible, its virtual modeling [6,10].

AR technology serves as a modern means of visualization and navigation, both during preoperative planning and during surgery [11]. It represents the integration of digital information (most often visual) with real-world objects using specialized technical devices, such as augmented reality glasses [12,13].

AR technology has been utilized in medicine since the 1980s. Prior to this, it was employed in teaching the management of complex technical equipment. The current stage in the medical application of AR is characterized by its transition from being merely a training and preoperative planning tool to a tool for intraoperative navigation. The introduction of AR technology in the surgery of pelvic tumors is an example of the application of information technologies in medicine, akin to software used for predicting disease progression [14].

There is a range of publications in the literature devoted to the use of AR technology in the stages of preoperative preparation and planning across various fields, including cardiac surgery, urology, neurosurgery, and maxillofacial surgery. Publications also discuss the use of AR in colorectal cancer surgery, including cases of colorectal tumor recurrence and recurrent tumors of the pelvic organs (RTPO) [15,16,17,18].

However, the efficacy of existing methods of applying AR technology and the usefulness of digital imaging of tumors of various localizations during surgical procedures are actively debated in the scientific community and have not yet been conclusively shown [19]. Researchers generally agree on the need to accumulate and evaluate the experience of using AR in specific areas of surgery.

Therefore, the opportunities that AR provides at the stage of preoperative planning and during the surgical intervention itself are highly sought after in the surgery of locally advanced primary tumors and RTPO. This underscores the need for continued clinical studies.

Objective: To investigate the potential applications of AR technology during surgical procedures in the treatment of patients with locally advanced primary and recurrent pelvic tumors.

Tasks:

1. To develop and test an algorithm for utilizing AR technology in simulating surgical interventions for patients with locally advanced primary and recurrent pelvic tumors.
2. To assess the technical possibility of applying AR technology during surgical procedures in treating patients with locally advanced tumors of the abdomen and pelvis.

## 2. Materials and Methods

The study was conducted at the Naval Surgery Chair Clinic of the Kirov Military Medical Academy and the Department of Coloproctology of the I.I. Janelidze Emergency Research Institute by a study team (ST). The ST included the authors of this paper, along with a wide range of employees from both clinics who contributed to this work. The study received approval from the local ethics committee: the Independent Ethics Committee at the Federal State Budgetary Military Educational Institution of Higher Professional Education “Military Medical Academy named after S. M. Kirov” (reference number 247 dated 26 January 2021).

A step-by-step algorithm for the use of augmented reality (AR) technology in the surgical treatment of patients with locally advanced primary tumors and recurrent tumors of the pelvic organs (RTPO) was developed and tested in all patients included in the study.

The development of the algorithm was based on the spiral model, a well-established methodology in software development. This model emphasizes iterative cycles, where each iteration involves a thorough evaluation and analysis of the algorithm, followed by necessary optimizations and enhancements. This approach was crucial for refining the AR technology used in the surgical treatment of patients. Every phase of the algorithm’s development underwent refinement, ensuring its effectiveness and reliability of this approach in a clinical environment. The process was not limited to technical adjustments; it also integrated insights and feedback from the multidisciplinary team, which included experts such as surgeons, oncologists, and engineers. This collaborative and iterative refinement was key in adapting the algorithm to meet the complex demands of the operating room.

The technical foundation of the study consisted of an augmented reality hardware and software complex (HaSC-AR-v1), which included a personal computer and AR glasses “Microsoft Hololens 2” using optical markers tracking [20] as a positioning approach. Additionally, a number of marker fixation system options were utilized, combining the properties of an X-ray contrast marker [21] and marker attachment that allowed fixing of a marker with a tracking image on a patient for integrating a virtual image into the operating field during surgery [22]. The integrated topographo-anatomical model of the patient was constructed by a multidisciplinary team through segmentation of preoperative CT data in the open software “3D Slicer 5.2” and adapted for use during the surgical procedure using software created by the authors.

The clinical part of the study involved 11 patients who were in receiving inpatient treatment at the clinic of the Naval Surgery Chair and the Coloproctology Department of the I.I. Janelidze Emergency Research Institute from November 2021 to August 2022. All patients provided informed consent to be included in the study. They underwent a comprehensive preoperative examination according to national recommendations for the treatment of pelvic cancer. Locally advanced primary tumors and RTPO were confirmed in all cases, including morphological verification. CT angiography of the pelvis with a combined X-ray contrast marker was used as an additional diagnostic method.

The main data about the patients are summarized in tables for analysis. Numerical quantitative data were subjected to statistical processing as they displayed an abnormal distribution. Data are presented with the median, the 25th, and 75th percentiles.

## 3. Results

### 3.1. IT-Results

**Figure 1:**
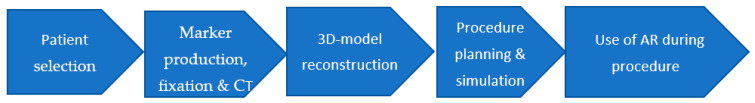
Step-by-step algorithm of augmented reality use in surgery (AlARUS).

To standardize the approach and unify the technique of AR in surgery, the study team (ST) developed and tested a step-by-step algorithm for augmented reality use in surgery (AlARUS), which comprises five consecutive stages (Figure 1).

In this study, we were able to fully implement the AlARUS algorithm in eight patients (72.72%). In three cases (27.27%), a modified, shortened version of AlARUS was utilized. This adaptation was necessary because of specific clinical challenges: one patient experienced tumor invasion into the hip joint, and two patients had distant, unresectable metastases. These conditions precluded the possibility of both full radical surgical intervention and the complete application of the AlARUS algorithm.

### 3.2. Patient Selection

Drawing from the experience of using AR technology in neurosurgery and maxillofacial surgery [19], we recognize certain conditions as necessary for accurately aligning the virtual model with the intraoperative scenario. These conditions include: the structures represented in the 3D model are static, situated within a relatively enclosed space with bone support, and the positioning of the body has minimal impact on the spatial relationship of the structures of surgical interest. Locally advanced primary and recurrent tumors of the pelvic organs typically exhibit these characteristics. Consequently, this makes the application of AR technology possible and effective in the surgical treatment of this patient category.

### 3.3. Marker Production, Fixation, and CT

At this stage, the study team (ST) utilized several variants of fixation systems they developed.

**Figure 2:**
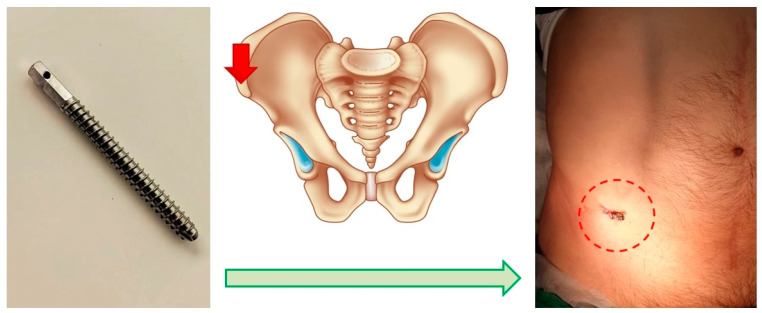
Schematic of the Invasive Fixation System. Screw for marker positioning (**left**), planned point for screw insertion (**middle**), post-installation view (**right**).

The first was an invasive system, comprising a titanium threaded pin implanted percutaneously into the anterior superior spine of the ilium. This pin, notable for its necessary contrast, is immovably fixed in the bone structures. It serves to attach the marker [19], facilitating the comparison of the topographic and anatomical model during surgery (Figure 2).

Additionally, a non-invasive marker fixation system was employed. This system’s marker features a plastic base with three magnetic supports. These supports adhere to the skin via magnetic interaction with magnetic parts of a special unit, which is affixed to the skin using a sticky film. These magnets also serve as radiopaque tags for computed tomography.

Following the implantation of the pin or the application of magnets, a CT scan of the abdomen and pelvis with intravenous contrast was performed.

### 3.4. 3D-Model Reconstruction

One of the primary objectives of this stage of AlARUS was to generate an anatomically precise, personalized 3D model (3DM) that is visually accessible and suitable for display on the surgeon’s AR glasses during the procedure. The creation of the 3DM was carried out using the open-source software 3D Slicer by a multidisciplinary team. This team included a surgeon, oncologist, radiology specialist, and other relevant specialists (urologists, gynecologists, vascular surgeons, neurosurgeons), as well as an engineer. The process involved several directions and steps (Table 1). The primary data for creating the 3DM were CT results, presented as DICOM images. Additionally, MRI data were utilized for a detailed assessment and clarification of the nature of the local spread of malignant pelvic tumors.

**Figure 3:**
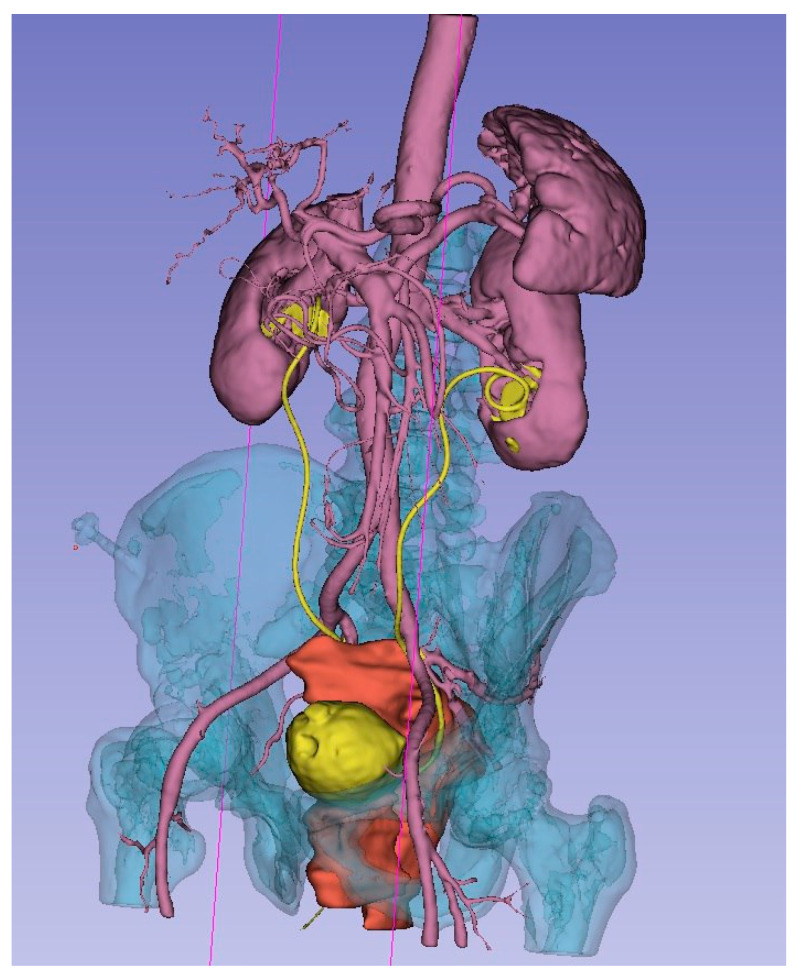
3D model of local advanced rectum tumor (red), bladder and ureters (yellow), arteries, kidneys, spleen (rose), bone (blue).

As a result of the concerted efforts of each participant and the team as a whole, a 3D model was meticulously created. This model accurately represents the bone structures of the pelvis, bladder, uterus and its appendages, ureters, major vessels (including the aorta, inferior vena cava, common iliac vessels, external and internal iliac vessels, and their tributaries), and the malignant tumor. Each organ and structure involved in the malignant process was segmented individually for detailed analysis. To enhance visual perception, each element of the 3D model was highlighted in a distinct color. Separately, the planes of tumor ingrowth into organs, the proposed sites for vascular ligation, the levels of ureteral resection, and the boundaries of the planned optimal plane of resection were all meticulously annotated (Figure 3).

### 3.5. Procedure Planning and Simulation

Procedure planning and simulation were performed through a multidisciplinary consilium. Each patient included in the study was evaluated during a consultation involving a surgeon, oncologist, radiology specialist, and other related specialists from the study team (ST). Urologists, gynecologists, and vascular surgeons were also invited as needed. In two cases, the involvement of a neurosurgeon and an orthopedist was necessary to determine the optimal approach for sacral resection. During these consultations, all participants had the opportunity to review the patient’s 3D model (3DM) and all relevant medical documentation.

### 3.6. Using Augmented Reality during Procedure

**Figure 4:**
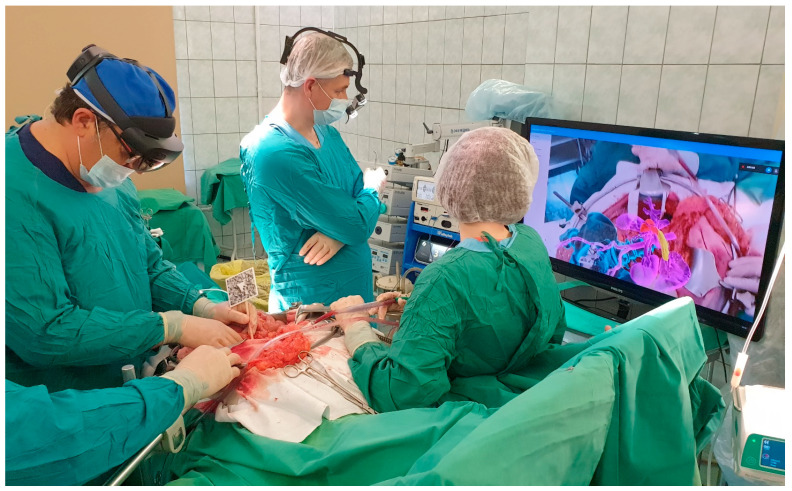
Augmented reality use during procedure.

All surgeries were conducted under general combined anesthesia. Following a median laparotomy and initial exploration, a special X-ray contrast marker was affixed to the pin previously installed in the iliac spine or attached to the skin-applied magnets. The operating surgeon used AR glasses ‘Microsoft Hololens II’ for intraoperative navigation. The software developed by the study team (ST) loaded a virtual image of the 3D model into the memory of the AR glasses and projected it onto the display system. Simultaneously, the software aligned the 3D model with the marker, correctly positioning it within the operating field. The image displayed in the AR glasses was also displayed on an additional monitor for the convenience of the operating team and to facilitate possible modifications to the operation plan (Figure 4).

Thanks to the AR navigation system, the margins for optimal tumor resection, levels for ligation of the main vessels, points of organ intersection, and levels for ureteral and other anatomical structures’ resection were refined or corrected. The median time spent using the AR glasses during a single surgical procedure was 18.5 min, with a range of 14.5 to 27.75 min.

### 3.7. Clinical Results

A total of 11 patients were enrolled in the study, which were divided into two groups. The first group consisted of five patients with locally advanced primary tumors of the pelvic organs. The second group comprised six patients with verified recurrent tumors of the pelvic organs (RTPO) (Table 2 and Table 3).

**Figure 5:**
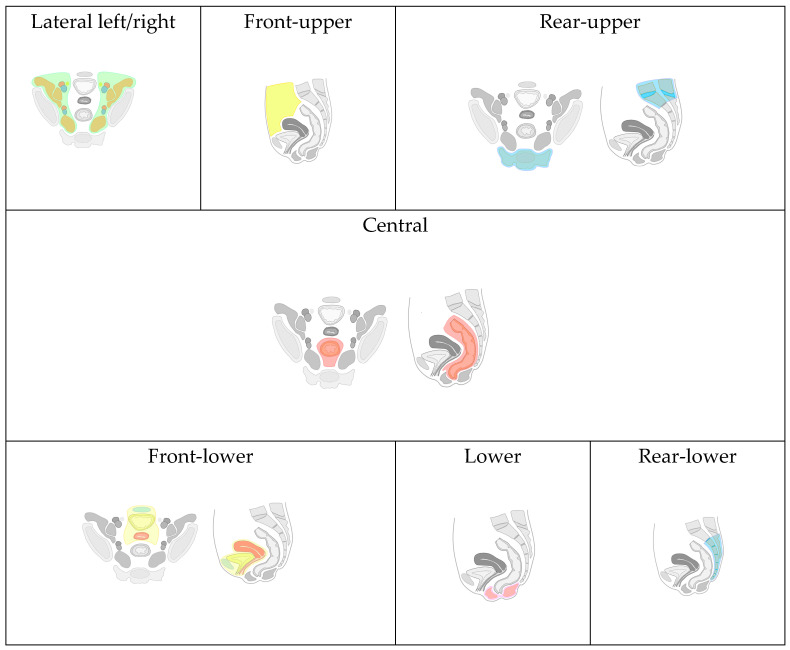
Classification of recurrence tumors of pelvic organs.

Due to the lack of a universally accepted classification for recurrent tumors of the pelvic organs (RTPO) and the varied involvement of anatomical structures and organs in malignant lesions, we have developed and implemented a map. This map comprehensively details the anatomical locations and the extent of local spread of RTPO (Figure 5). The purpose of this map in our study is to structure presented surgical cases and to demonstrate wide applicability of AR technology across different surgical scenarios.

Based on the proposed classification, eight types of local RTPO were identified, categorized by specific organ and anatomical formation invasions:

Front-Upper (FU) Type: Includes lesions of the jejunum, iliac colon, sigmoid colon, large omentum, and anterior abdominal wall (highlighted with yellow color in Figure 5).

Front-Lower (FL) Type: Encompasses invasions of the bladder, urethra, seminal vesicles, prostate gland, uterus, cervix, fallopian tubes, ovaries, vagina/vaginal stump, and the pubic joint (highlighted with blue, yellow, red colors in “Front-lower” frame in Figure 5).

Central (C) Type: Involves invasion into the rectum, its stump or anastomosis, mesorectal tissue, and presacral fascia (highlighted with red color in “Central” frame in Figure 5).

Rear-Upper (RU) Type: Covers lesions of the sacrum above the second vertebra. In contrast, the rear-lower type involves the second sacral vertebra (S-II) and/or coccyx (highlighted with blue color in “Rear-upper” frame in Figure 5).

Lower (L) Type: Involves the pelvic floor muscles and/or the perineal scar(highlighted with pink color in “Lower” frame in Figure 5).

Lateral Left (LL) and Right (LR) Type: Includes invasion of the ureters, obturator nerve, piriformis muscle, and iliac vessels (common, external, and internal arteries/veins), (highlighted with green and brown color in “Latera left/right” frame in Figure 5, vessels are highlighted with blue and red).

Combined Relapse: The identification of two or more types in one patient indicates a combined relapse, with the specific types involved being noted.

In this study, combined types of tumor recurrence were registered in all six clinical cases. Notably, the posterior-upper type of RTPO (sacrum above S-II) was not observed in the presented sample (Table 4).

All patients in the study exhibited tumors involving more than one anatomical structure. The minimum number of involved structures was two, while the maximum was seven. The characteristics of the involved structures are presented in Table 5.

**Figure 6:**
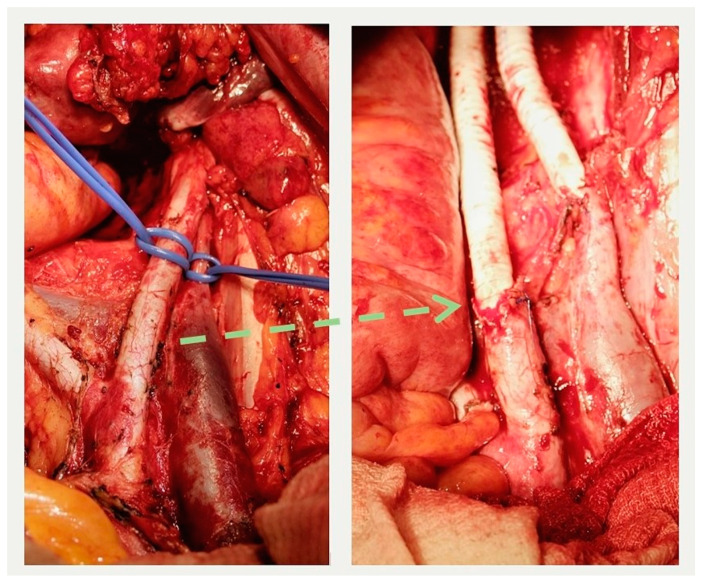
External iliac artery and vein before resection (**left**) and after reconstruction (**right**). The interrupted arrow indicates the proximal anastomosis between the resected iliac artery and the graft.

In the group of patients with primary tumors, three surgical interventions of varying volumes were performed: combined total supralevator evisceration of the pelvis, combined posterior supralevator evisceration of the pelvis with resection and synthetic graft reconstruction of the right external iliac artery and vein (some operation field views are presenting in the Figure 6), and combined resection of the bladder. The advanced invasion of the tumor into the pelvic bone structures, including the involvement of the hip joint, was the reason for opting out of radical surgery in one case. In another clinical case, the presence of distant, unresectable metastases precluded the possibility of performing radical surgery.

In all three patients who underwent radical operations, AR technology was employed, including the use of DR during surgery. In each case, a highly accurate alignment of the 3D model with the operating field was observed. The use of markers did not present any difficulties for the surgical team. Complete concordance between the 3D model and the anatomical and pathological structures of the abdominal cavity and pelvis was consistently noted. There were no unexpected ‘surgical surprises’ or findings that had not been previously documented in the 3D model. Surgeons subjectively reported high levels of comfort during the operations. Furthermore, there were no complications or adverse events associated with the use of AR technology.

In the group of patients with recurrent tumors of the pelvic organs (RTPO), five radical surgeries of varying volumes were performed: two combined infralevator total pelvic eviscerations with distal sacrectomy, one supralevator total pelvic evisceration, and two combined anterior pelvic eviscerations. One patient underwent a palliative colostomy due to rapid tumor growth, the presence of distant, unresectable metastases, and the progression of colonic obstruction.

AR technology was utilized in all five patients who underwent radical surgery, including its application during the surgical procedures. In every instance, there was an accurate correlation between the 3D model and the actual operating field. The use of markers did not result in any inconvenience. Moreover, there was consistency between the 3D model and the anatomical and pathological structures in all cases. Notably, there were no ‘surgical surprises’ or findings that were not already documented in the 3D model. Surgeons subjectively reported a high level of comfort during the operations. Importantly, there were no complications or adverse events associated with the use of AR technology.

For the eight patients in whom AlARUS was implemented, the median duration of surgery was 202.5 min (range: 117.5 to 282.5). The median blood loss was 300 mL (range: 187.5 to 625). The median length of hospital stay was 21 days (range: 17.75 to 27.75). Complications were encountered in three patients (37.55%) and included perineal suture skin necrosis, ascending pyelonephritis, and hospital-acquired pneumonia. No hospital mortality was reported. Postoperative specimen morphology indicated that a negative resection edge (R0—resection) was achieved in all eight patients. All patients were discharged for follow-up and systemic anti-tumor therapy.

Overall, AlARUS was applied in 11 patients, and 3D model (3DM) construction was performed for each. The clinical situation allowed for the use of AlARUS during surgery in eight of these patients (72.72%). In all these cases, the planned surgical interventions were executed according to the plan created by the multidisciplinary team, accounting for 72.72% of the entire cohort.

## 4. Conclusions

Step-by-step algorithm for using AR in surgery. The algorithm developed and tested for the use of augmented reality technology in surgical interventions for patients with locally advanced primary and recurrent pelvic tumors can be characterized as both reproducible and efficient. This comprehensive algorithm includes stages such as patient selection, marker production and fixation, CT scanning, 3D model creation, procedure planning and the use of AR during surgery. This approach for procedures for patients with locally advanced primary and recurrent tumors of the abdomen and pelvis can lead to a potential increase in safety and enhanced radicalism in treatment. This advancement marks an improvement in the surgical management of these complex cases.

Combined surgical procedures in patients suffering from locally advanced primary and recurrent pelvic organ tumors present a contemporary challenge in the field of modern surgical oncology. The significant surgical trauma, associated perioperative risks, the imperative need to achieve negative surgical resection margins, and the inherent technical complexities in pelvic procedures all underscore the necessity for improved therapeutic and diagnostic strategies for treating this patient population. Therefore, the most promising avenue for treatment, in addition to the development of novel surgical approaches and optimization of intensive care protocols, is the adoption of a multidisciplinary approach to tactical decision-making, tailored to individual patients. One of the particularly promising solutions, supported by a wealth of literature data, involves the development and clinical application of innovative imaging technologies, notably AR.

An analysis of 11 cases involving patients with locally advanced primary and recurrent pelvic organ tumors reveals several noteworthy conclusions:

1. This technology can potentially enhance the efficiency of preoperative diagnosis and offers a comprehensive assessment of the local and regional extent of the lesions.
2. AR establishes the groundwork for better surgical planning by a multidisciplinary team, paving the way for innovative surgical modeling technology. The detailed visualization and 3D modeling of locally advanced primary and recurrent tumors, along with the identification of their points of attachment to surrounding organs and vital anatomical structures within the pelvis, not only facilitate the planning of the surgical procedure but also enable the formulation of a precise sequence of surgical maneuvers aimed at safely mobilizing the organ complex slated for resection. Furthermore, it aids in marking the configuration of the dissection plane, ensuring the attainment of negative surgical margins with a high degree of reliability.
3. The utilization of advanced imaging technologies empowers the surgical team to collaboratively engage with the 3D model developed during surgery, facilitating multidisciplinary planning. This aspect finds strong psychophysiological justification and significantly contributes to the success and seamless progression of the surgical procedure.

Preliminary Assessment and Future Research: Initial experiences indicate the benefits of AR in preoperative procedures for patients with locally advanced primary and recurrent pelvic tumors. However, the potential of AR use during surgery requires further exploration. While preliminary assessments suggest increased safety in multivisceral resections and pelvic eviscerations, a definitive conclusion on AR’s role in surgery and its impact on outcomes is yet to be drawn. Given its innovative nature, AR technology promises to improve complex pelvic organ resections, necessitating further study and clinical application. The focus should be on developing and refining AR technology for modeling combined surgical interventions, enhancing intraoperative methods, and developing metrics to accurately assess the efficiency [23] and feasibility [24] of preoperative planning and surgical interventions with AR. Increasing the sample size and incorporating efficiency measurements in future research is essential.

## Figures and Tables

**Table 1:** Creation of the 3D Model by a Multidisciplinary Team.

	Multidisciplinary Team			
	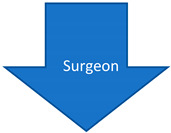	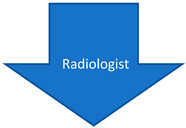	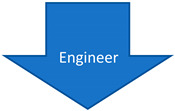	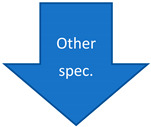
	Directions for Creating the 3D Model			
Steps for Creating 3D Model	Diagnosis formation	Details of diagnosis based on CT data	Case-specific software correction	Profile diagnosis
	Procedure zone anatomy correction	Marking of anatomical structures	Creation of case-specific 3D models	Profile-specific 3D model correction
	Procedure scope presentation	Determination of critical surgical points	Marking critical surgical points	Planning profile maneuvers
	Details of maneuvers and anatomy	Details of critical surgical points	Simulation of dissection planes	Decision on reconstruction profile
	Reconstructive plandecision	Emphasis on case-specific anatomy	Simulation of reconstruction	Supplementing the profile-specific 3D model

**Table 2:** Clinic characteristic of primary tumor group (*n* = 5).

Patient ID	Gender	Age	Tumor-Source Organ	TNM
1	f	62	Ovarium	T3cN0M1
2	f	42	Cervix Of Uterus	T3bN0M0
3	m	55	Bladder	T4aN2M1
4	m	57	Rectum	T4bN1M0
5	m	45	Bladder	T4bN3M0

**Table 3:** Clinic characteristic of RTPO group (*n* = 6).

Patient ID	Gender	Age	Tumor-Source Organ	Primary Surgery Type	TNM
1	m	53	Rectum	APR *	T3cN1aM0
2	m	67	Rectum	APR *	T3N1aM0
3	f	43	Cervix Of Uterus	Extirpation of uterus	T1bN0M0
4	f	46	Cervix Of Uterus	Extirpation of uterus	T2aN1M0
5	m	52	Bladder	Cystectomy	T4aN1M0
6	f	42	Corpus Of Uterus	Hysterectomy	T2aN0M0

* APR—abdomino-perineal resection.

**Table 4:** Frequency of different tumor recurrence types.

Type of Recurrence	Number	Frequency, per 100 Cases
Front-upper (FU)	3	14.29
Front-lover (FL)	5	23.81
Lover (L)	3	14.29
Central (C)	4	19.05
Rear-upper (RU)	0	0.00
Rear-lower (RL)	2	9.52
Lateral-left (LL)	2	9.52
Lateral-right (LR)	2	9.52
Total:	21	100

**Table 5:** Frequency of involving different pelvic organs in recurrence tumor.

Organs and Anatomic Structures	Numbers	Frequency per 100
Bladder	6	14.29
Ureter	4	9.52
Prostate, vesicles	2	4.76
Vagina (vagina stump), uterus with appendages	3	7.14
Rectum (rectum sump)	4	9.52
Sacrum (lower than SII), coccyx	2	4.76
Perineal muscles and scars	2	4.76
Abdominal wall	2	4.76
External iliac artery	5	11.90
Internal iliac artery	3	7.14
Pelvic bones structures	2	4.76
Other *	7	16.67
Total:	42	100%

* Small and large bowel, peritoneum, omentum, spleen, hip joint, iliac bones.

## Data Availability

Data are contained within the article.
